# An efficient blockchain-based framework for file sharing

**DOI:** 10.1038/s41598-024-69011-4

**Published:** 2024-08-03

**Authors:** Wanzong Peng, Tongliang Lu, Wenju Peng, Zhongpan Wang

**Affiliations:** 1 School of Cyberspace Science, Harbin Institute of Technology, Harbin, 150001 China; 2 PLA78156, Chongqing, 400000 China; 3 Department of Engineering Physics, Tsinghua University, Beijing, 100084 China; 4 PLA31202, Guangzhou, 510000 China

**Keywords:** Computer science, Information technology

## Abstract

File sharing, being the foundation of the Internet, has traditionally relied on a centralized service architecture resulting in significant maintenance costs. Moreover, due to the lack of an effective file management system, instances of sensitive information going out of control and loss of confidentiality in file sharing have occurred frequently. In order to address the difficulty of tamper detection and the lack of supervision in the entire process of file transfer in the current Internet environment, this paper designs a blockchain-based system architecture for secure sharing of electronic documents. An efficient blockchain model is used in our framework, and with the help of distributed storage system and asymmetric encryption technology, file sharing can be controlled, reliable and traceable in the transfer process. Referring to existing consensus mechanisms, e.g., Delegated Proof of Stake (DPoS) and Practical Byzantine Fault Tolerance (PBFT), we propose a new consensus for efficient and secure file sharing. Our experimental results show that our framework can maintain a higher throughput than existing schemes

## Introduction

In recent years, the security of network files, especially confidential and sensitive information, has gained increasing attention. The traditional electronic file transfer process mostly uses Client/Server model, where data security is solely dependent on the capabilities of larger companies. This model entails uploading electronic files to specific servers for centralized management. However, it presents various challenges such as increased load on the central server, excessive overhead on system resources, and high deployment and maintenance costs. Additionally, since each server possesses its own storage device, it is difficult to ensure server reliability and data security. In order to overcome these problems, the concept of cloud storage was proposed and remains widely used, there are many large enterprises (e.g., Microsoft and Amazon) provide cloud storage services to individuals, businesses and communities. Cloud storage providers take responsibility for maintaining the storage servers and ensuring data security for users (both personal and corporate), thereby eliminating the need for users to maintain storage devices. Personal cloud storage platforms (e.g., OneDrive, Google Drive) enable individuals to back up and protect their data, with the added benefit of being able to access and share their stored files online from anywhere. However, cloud storage is not without its shortcomings. Most cloud storage services are provided by large enterprises, resulting in increased centralization of data compared to previous systems. Although this has enhanced efficiency, users remains a lack of full trust in large enterprises, as they may potentially abuse personal data and information.

So people want to build new model to share file: the P2P (peer-to-peer) file sharing system. Unlike C/S model, each node in the P2P system, which works as both a client and server, is on equal terms. Following the widespread adoption of Napster (a music file-sharing service), P2P file sharing programs came to have great popularity^1^. Napster allowed users to share digital music files such as MP3. Users can transfer files to each other by Napster, but Napster did not follow a pure P2P architecture since it required centralized infrastructure for indexing of published documents^2^. In 2001 Bram Cohen created BitTorrent, which is one of the most successful open P2P applications that has completely changed people’s habits of file transfer. Originally BitTorrent was based on a centralized server coordinating the interaction between peers, and the centralized server is named as tracker, which keeps track of all the peers. But BitTorrent later began to adopt more advanced technologies: distributed hash table (DHT), a distributed system for mapping keys to values. BitTorrent builds a truly decentralized network based on the Kademlia DHT^3^, there are not central servers and each device is free to join network as a server, or a client, or both. Compared with the traditional C/S model, P2P system can fully utilize idle network resources and achieve better load balancing. But the freedom brings hidden security risks. P2P network is applied on the premise of trust between users, but is vulnerable to illegal access and malicious attacks^4^, resulting in the disclosure of sensitive information. Therefore, it is essential but difficult to provide a high-performance and secure file sharing system.

The growth of the Internet of Things (IoT) has led to increased research^5^ on distributed information systems due to its distributed nature, and traceable distributed data sharing solutions have emerged in IoT^6^. With the popularity of cryptocurrency all over the world, blockchain technology has attracted tremendous interest from both academia and industry^7^ and applied in various fields, e.g., healthcare, Internet of Things (IoT), and cloud storage^8^. The decentralized nature and reliable security features of blockchain technology offer new perspectives on file transfer reliability. Leveraging this idea, we develop a more efficient file-sharing system that save server resource consumption.

The main contributions of this paper are as follows:

- A proposed efficient method for file sharing utilizes blockchain technology. By the method, the existing storage system (e.g., cloud storage platform, P2P system) is used to store file and the blockchain is only used to save information about file sharing.
- We build a blockchain with new framework, which contains two chains. The information of file is stored in the particular chain. We design a special data structure for file information, so we can reliably track the source and monitor the lifecycle of file.
- A new consensus is proposed, which groups nodes and conducts transactions efficiently. We demonstrate its feasibility through evaluation and our experiment results show that our framework is more efficient than existing frameworks.

## Related work

After the emergence of blockchain, people considered using it for cloud storage^9^. Initially, the application of blockchain for cloud storage was rudimentary and centralized, leveraging its inherent properties for enhanced security and integrity. However, this approach did not fully exploit the potential of blockchain’s decentralization. Then Benet created Inter Planetary File System (IPFS)^10^. IPFS is a distributed file system, and like blockchain, it is a P2P network run by multiple nodes. So many people begin to combine it with blockchain technology for file transfer. Chen et al.^11^ proposed an enhanced P2P file system scheme, improving IPFS’s block storage model with a zigzag-based storage solution, and employing blockchain to facilitate better coordination among nodes for efficient data exchange. Vimal et al.^12^ utilized Filecoin as an incentivization mechanism for content providers based on the integration of IPFS and blockchain technology. Subsequently, some schemes have sought to enhance IPFS with Hyperledger Fabric.^13,14^While these schemes improve file sharing security and reliability, they typically rely on existing blockchain systems like Ethereum or Hyperledger Fabric for implementation, which may lack efficiency.

There has been a growing interest in high-performance information sharing blockchain in the field of IoT. Dorri et al.^15^ proposed a lightweight blockchain architecture for IoT. Xu et al.^16^ proposed DIoTA, a decentralized ledger-based framework to authenticate IoT devices and data generated from them. And people are also beginning to use the next generation blockchain for data sharing: Directed Acyclic Graph (DAG) Distributed Ledgers, e.g., IOTA^17^, Nano^18^. The DAG structure allows parallel validation of transactions and reduces the consumption of transaction. So DAG distributed ledgers can establish more efficient and scalable file share system, such as FileDAG^19^.

**Figure 1:**
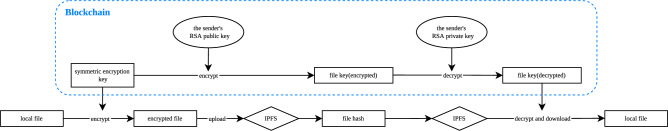
The process of file sharing method.

## Method and functions

The main aim of our system is to share file safely. So we design a complete method to share file, it is depicted in Fig. 1. It can be divided into two parts: in the first part, file is encrypted and stored in IPFS; in the second part, information of file and user is stored in blockchain. We define two main functions to finish file transfer.

### Upload

The user encrypts the local file with a randomly generated symmetric encryption key (as “the file key”) and uploads it to IPFS to obtain the file hash (i.e. the IPFS content identifier, which is used to obtain the file). Of course, we can also use other storage platform (e.g., cloud storage platform). Each user need generate a “blockchain wallet”, simplified as an asymmetric key here. The user’s public key encrypts the file key, which is then stored in the blockchain transaction alongside the file hash and relevant information. The process is depicted in Algorithm 1.

**Algorithm 1:**
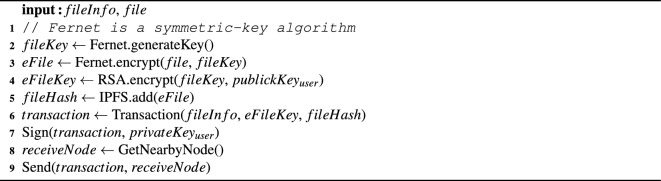
Upload file.

### Download and transfer

During the download process, the file owner retrieves the file hash and key from the blockchain, downloads the file from IPFS through the file hash, and decrypts the file key with a private key to decrypt the file. During transfer, the file key is first decrypted using the owner’s private key, and then encrypted using the recipient’s public key. Finally, the encrypted file key and file hash are stored in the transaction. Essentially, the file hash serves as an equivalent representation of the file on the blockchain, facilitating secure retrieval and transfer of the file. The process is depicted in Algorithm 2.

**Algorithm 2:**
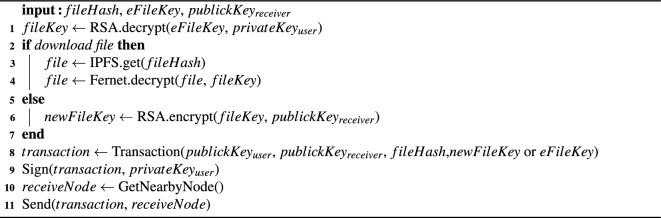
Transfer file.

## Framework

For efficient transfer, we need a novel blockchain that can achieve high concurrency and security. Therefore, we borrow from traditional blockchain and consensus algorithms widely used in cryptocurrency systems, and propose our framework. We utilize a two-chain structure for optimal performance. The File Transaction Chain is designed with specialized data structures to efficiently store transactions and maintain data integrity, while the File Info Chain functions as a traditional blockchain to safeguard the security and stability of the system.We simply discuss one kind of nodes: full nodes, which validate transactions and blocks, ensuring they adhere to the network’s consensus rules. Full nodes stores a complete copy of the two-chain structure blockchain and relay new transactions and blocks to other nodes in the network. In practical situations, there will also be lightweight nodes^20^ in the system.

### File transaction chain (FTC)

**Figure 2:**
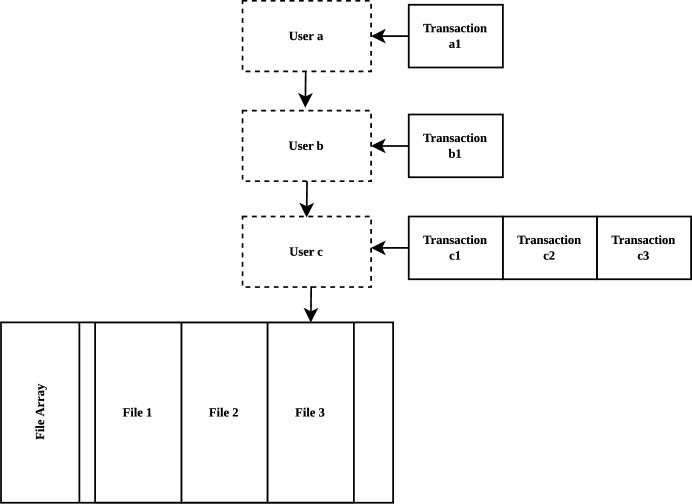
The data structure of File Transaction Chain.

This chain handles transactions about file and provide information of file to user. When a transaction is validated, it is stored in the following structure: To facilitate traceability, the file is used as a root block to form an array; the user group is attached to the corresponding file, where each user points to the user who shares the file to him to form a chain structure; transactions are sorted chronologically and attached to the corresponding users as an array. The overall structure is shown in Fig. 2. Main information is stored in the following sections:

- File: file name, file hash, created time and other related information.
- User: user’s public key and the encrypted file key.
- Transaction: address of both parties, transaction type, timestamp, transaction information, signature of validation node group, signature of transaction creator and transaction hash value.

### File info chain (FIC)

This chain functions similarly to an traditional blockchain, which is used to manage. It stores system information such as voting, node reputation, and efficiency. These information are stored in transactions, and each block contains: header (previous block hash, timestamp, nonce), Merkle root^21^, transactions. In summary, the data structure adopts a traditional chain structure.

### Normal-case operation

There are 5 main steps for the whole system’s lifecycle. These steps also show the consensus. We propose the new consensus based on the concepts of sharding and PBFT. The lifecycle is shown in Algorithm 3.

1. **Vote to select the leadership group.** Nodes vote based on efficiency and reputation of each node. The number of votes that can be used by the node is determined by the rating of the nodes on FIC (File Info Chain) and each node has equal right in the first time. Nodes broadcast the voting results as a transaction to all nodes, and after obtaining all voting results, each node calculates the node ranking. The top 1/5 nodes are selected as the leadership group.
2. **Divide node groups by the leadership group.** The members of the leadership group rotate as chair according to the ranking order. Based on the information blocks on FIC, each node is rated and scored. The chair divide nodes into 6 groups (the number of groups is adjusted reasonably according to the node size and transaction volume) and make sure that each group has similar total score.
3. **Create block on FIC.** The chair packages the information about voting results, node grouping and other system (e.g., efficiency information, reputation information) as transaction, then chair requests the transaction according to the PBFT algorithm^22^. When 2/3 of the leadership group nodes confirm the result, blocks will be created on FIC. If more than 1/3 of the members do not agree with the grouping, they need to go back to the second step until a grouping is formed.
4. **Conduct transactions.** Nodes in each group form an independent peer-to-peer network, and adjacent groups establish P2P network channels to form a ring network structure. When one user want to upload or transfer file, he initiates transaction on FTC in the node of previous group and send it to a random node in the next group. After receiving the transaction, the receiving node broadcasts the transaction within the transaction processing group. After more than 2/3 of the nodes in the group have validated and signed, the receiving node attaches all signatures to the transaction and broadcasts it to its group and the leadership group. The leadership group forms a P2P network channel with each group. After receiving and validating it, the leadership group nodes broadcast the transaction to each group, and each node inserts the transaction into the transaction array of the user in chronological order based on the corresponding files on File Transaction Chain. Any transaction that have failed validation in 1/3 of the validation nodes will be discarded and notified to the transaction initiator and leadership group.
5. **Supervise transactions.** The leadership group evaluates the efficiency of each node based on the speed of transactions, initiates transactions containing efficiency information on FIC, randomly selects transactions for validation and labels transactions based on the results, and initiates transactions containing node reputation information. These transactions will be validated by the leadership group in the next time of grouping and the production blocks will be added to FIC.

Loop steps 4-5, and repeat steps 2-3 every 10 minutes. After rotating as the chair at all leadership group nodes, start from step 1 again.

**Algorithm 3:**
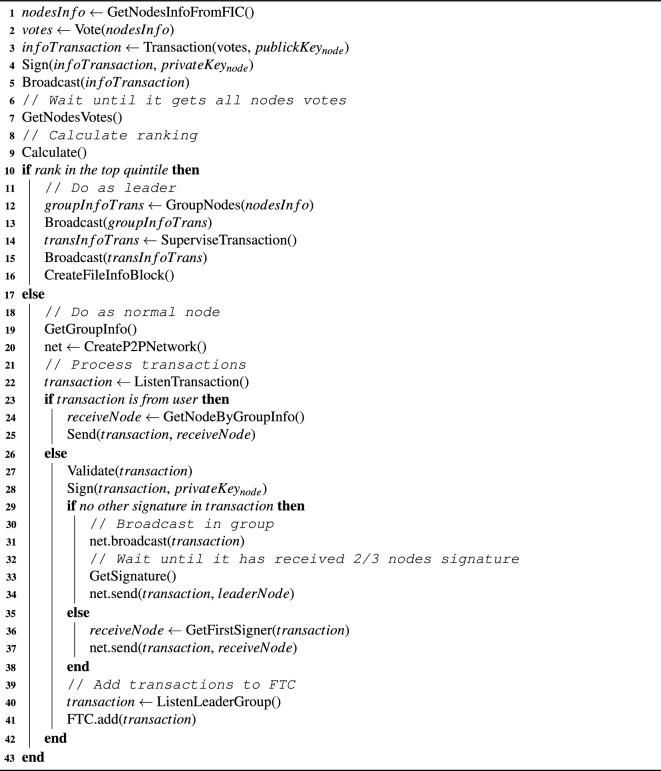
Nodes lifecycle.

## Security analysis

In our framework design, security is an important standard. Our framework runs safely and reliably through the use of cryptography and decentralization. We need to strive for efficiency while ensuring security. Therefore, we do not conduct thorough security audits and only analyze the security of this scheme for common security threats in the blockchain.

*Double spending attack* In normal blockchain system, attacker waits for specific conditions and uses cryptocurrency twice or more.This type of attack poses a significant threat to the integrity and security of blockchain systems by undermining the fundamental principle of cryptocurrency. But in our system, the crypto assets is file, owner can copy and tranfer file to anybody, there is no risk of double spending. The key aspect is to secure ownership through signature in our system, as files will not be “consumed”.

*Replay attack* When the user requests a transaction, attacker listens in and steals user’s information. Then attacker can send the same transaction again, and even modify transaction’s key information to make the file stolen. To prevent this attack, the communication between nodes is encrypted by one-time key pair in our system, and each transaction is created with a timestamp and an expiration time. Nodes within the system are configured to promptly identify and reject any transaction that is outdated or exhibits suspicious characteristics, such as duplicate timestamps or users, preventing potential security breaches.

*Impersonation attack* Attacker impersonates a legitimate user in order to gain access. Our framework uses RSA algorithms to create user’s wallet and key length is 2048 bits, which can provide enough security strength^23^. This approach emphasizes the need for utilizing advanced cryptographic techniques in blockchains. While more intricate encryption algorithms and longer key offer increased security, they may also result in higher performance degradation. Therefore, it is crucial to consider appropriate algorithms that can achieve a balance between security and performance based on the specific environment in which system is implemented.

*Sybil attack* Attacker subverts the reputation system of a peer-to-peer network by creating a large number of pseudonymous entities, using them to gain a disproportionately large influence^4^. Proof of Work (PoW) consensus does not depend on the number of nodes, Sybil attack can only cause limited damage to it and it is difficult to influence the entire blockchain network. But this type of attack is very dangerous to the consensus that run with voting (e.g., DPoS). To our consensus, if the attacker controls 2/3 nodes of a group, he can forge transactions. Due to the fact that node groups are divided by the leadership group based on node reputation and efficiency ratings, attacker need to control at least $\frac{2}{3\beta }$ nodes ($\beta$ is the number of groups) without a record of wrongdoing and then the attacker can control a group to create fraudulent transactions. But it is not enough to just fake transactions, the leadership group can determine whether the transaction is abnormal and record the malicious behavior by checking the hash and signature. So the fair election of the leadership group is an important guarantee for security. In order to measure the participation and credibility of the nodes and give different voting rights to the nodes, in this scheme, the effective transaction volume of the nodes is selected as the equivalent substitute of the computing resources of the nodes, and the reputation record is used to score the nodes. Unless the 51% attack is realized, the selection of the leader group is safe.

*51% attack* Attacker shows malicious behavior, such as tampering with transactions and forging blocks, by controlling 51% of the computing power of the entire network^24^. In our framework, if attacker controls 51% of the computing power, he can get enough right of voting to select the leadership group and control the entire system. This attack is challenging to defend against. But this is difficult to achieve in a large blockchain, the best prevention method is to establish a sufficiently large blockchain.

## Evaluation

To assess the progressiveness of this framework, we need to analyze the time consumption. The total process times (from the beginning of voting to the next revoting) between two different leadership group can be analyzed in two aspects: communication consumption and calculation consumption.

### Communication consumption

When a large number of nodes are evenly distributed in a network and there is no congestion caused by broadcasting, the average communication time RTT is considered as a fixed value. There are two types of communication consumption.

1. Vote and divide. Each node needs to broadcast its own voting results to all nodes, and the chair will divide nodes and broadcast once, requiring a total of 2 RTT.
2. Process and supervise transactions, create block on FIC. When each group conducts transaction processing, the transaction is initiated, and the receiving node receives it and broadcasts it to all nodes within the group for signature. Then, each node sends result to the receiving node for integration, and the receiving node broadcasts signed transaction to the group and leadership group, requiring a total of 4 RTT. While other groups conduct the transaction, the leadership group conducts transaction supervision. In extreme cases, all leadership group nodes record, validate, and broadcast the results to all nodes, with a broadcast time of 1 RTT. FIC block generation adopts the PBFT algorithm, which takes 5 RTT in 5 stages. The leadership group took a total of 6 RTT, which is longer than transaction processing.

Overall, in the lifecycle of a leadership group, each node mainly spends time processing transactions. In part 1 some individual communications consume less time and have fewer occurrences, and communication consumption is mainly considered in part 2.

### Calculation consumption

If we have a total of *n* nodes, $\gamma$ leadership group nodes, $\alpha$ transactions and $\beta$ groups, we can get algorithm complexity of the entire system.

1. Leadership group management. Dividing node requires iterating over blocks of FIC and scoring each node, with a complexity of $\textrm{O}\left( n\right)$. The time consumption of the transaction supervision part is linearly related to the number of transactions, and in the extreme case, $\gamma$ leadership group nodes record and validate $\alpha$ transactions with a complexity of $\textrm{O}\left( \frac{\alpha }{\gamma }\right)$. Each transaction can be completed by iterating over the transaction chain once per node, and the time consumption can be ignored. The block generation adopts the PBFT algorithm with a complexity of $\textrm{O}\left( \gamma ^2\right)$. We set average coefficient as $C_1$, the calculation consumption of the leadership group is:
  1 $$\begin{aligned} T_1=C_1 \times \left( \frac{\alpha }{\gamma }+n+\gamma ^2\right) \end{aligned}$$
2. Transaction processing. $\beta$ groups are conducted simultaneously, and the transaction is validated and signed by all nodes within the group after being broadcasted by the receiving node. Then, each node sends it back to the receiving node for integration, with a complexity of $\textrm{O}\left( \frac{\alpha }{\beta } \frac{n-\gamma }{\beta }\right)$. We set average coefficient as $C_2$ and the transaction processing time of each group is:
  2 $$\begin{aligned} T_2=C_2 \times \left( \frac{\alpha }{\beta } \frac{n-\gamma }{\beta }\right) \end{aligned}$$

Generally, $\alpha$ is much greater than $\gamma$. Through analysis, it becomes evident that increasing the number of nodes in the leadership group results in a decrease in the number of transactions handled by each node within the group. Consequently, this reduces the time consumption while simultaneously enhancing the degree of decentralization. However, $\gamma$ increasing will result the number of group nodes $\frac{n-\gamma }{\beta }$ in a too small number, which cannot guarantee the credibility of the transaction; moreover, the leadership group nodes have lost their transaction ability, and users can only create transactions through other nodes. Due to the limited processing capacity of other nodes, excessive number of leadership group nodes can lead to transaction congestion. So we need to select the appropriate number of groups and leader nodes based on the total number of nodes to ensure the overall system is reliable and efficient.

Compare the main parts of $T_1$ and $T_2$, $\frac{\alpha }{\gamma }$ and $\frac{\alpha (n-\gamma )}{\beta ^2}$. Because $\gamma <n$ and $\beta$ can be ignored compared to $n\gamma$, so we can get (3) and (4).

3 $$\begin{aligned} {\beta ^2}+{\gamma ^2}<n\gamma \end{aligned}$$

4 $$\begin{aligned} \frac{\alpha }{\gamma }<\frac{\alpha (n-\gamma )}{\beta ^2} \end{aligned}$$

$C_1$ is mainly caused by the program for transaction validation and recording, $C_2$ is mainly caused by the program for transaction validation and signature, and the consumption of signature algorithms is much greater than that of recording algorithms. So $C_2>C_1$ and $T_2>T_1$.

## Experiment

We direct our attention toward the meticulous analysis of $T_2$ through a series of intricate system simulation experiments. In our experimental setup, we have established a network environment where each server encompasses multiple nodes operating within the confines of a local LAN, engaging in peer-to-peer communication built by the TCP protocol to achieve a state of seamless “zero-latency” discourse. To conduct these experiments, we employ a cluster of six computers, each equipped with an Intel Core i5 processor operating at 3.6 GHz, 16GB of RAM, Microsoft Windows 11 64-bit version, and a 500GB hard drive. Within each computer, there are a total of 30 nodes, each running on different ports, resulting in a cumulative count of 300 nodes. Out of these nodes, 60 belong to the leadership group, while the remaining nodes are divided into 8 separate normal groups.

**Figure 3:**
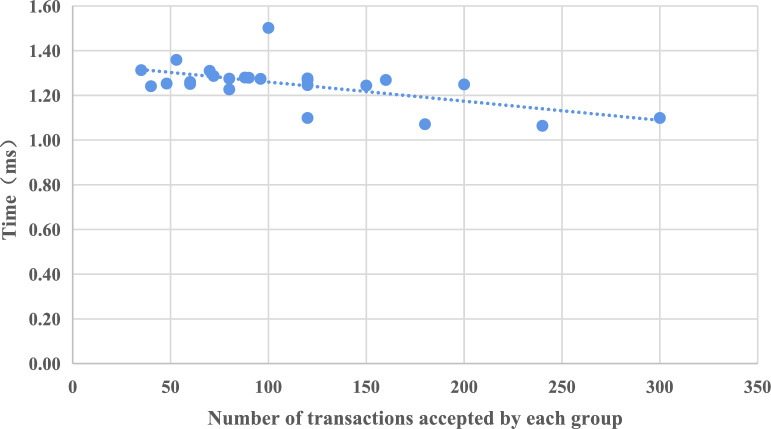
Average time consumption for each transaction.

We ensure that a fixed number of transactions are allocated to each normal group. Once all the transactions have been validated by the nodes in the leadership group, we proceed to gather and compute the average time spent on each individual transaction. The graphical representation of these results can be observed in Fig. 3. When the number of instantaneous transactions is below 300, the processing capacity of the group proves to be adequate, and the time consumption for the leadership group remains consistently stable. Therefore, as the number of transactions increases, there is a noticeable decline in the average time consumed per individual transaction.

**Figure 4:**
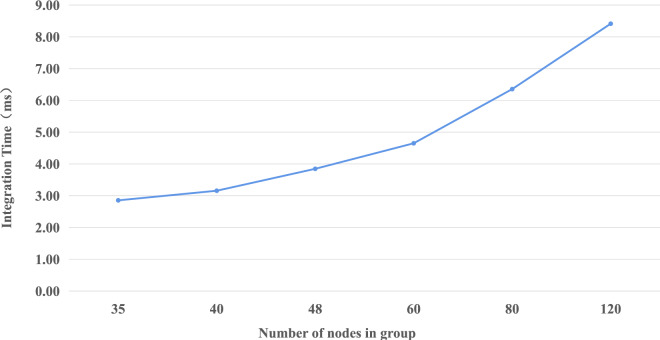
Integration time consumption for each transaction.

We change the number of nodes in each group to determine the impact of group size on transaction validation. After the number changes, each group requests 10 transactions. For each transaction, we collect integration time on the receiving node, which organizes signatures and broadcasts the result. The results, shown in Fig. 4, indicate that the integration time is short and increases slowly.

The impact of the number of nodes on transaction efficiency within a group is significant. The average number of transactions initiated by each node per second is TPS. To assess the processing capacity of groups with different sizes, we assign different TPS values and vary the number of nodes within a group. We measure the average time taken by each group to complete the requested transactions within one second. The resulting data is presented in Table 1. Blockage occurs when the total time consumed exceeds 1 second. From the table, it can be observed that when TPS is lower than 20, a group consisting of 40 nodes is an optimal choice.

**Table 1:** Transaction calculation time consumption.

TPS	Time consumption (second)					
	120 nodes	80 nodes	60 nodes	48 nodes	40 nodes	35 nodes
10	1.394	1.046	0.800	0.637	0.526	0.489
15	2.055	1.592	1.217	0.986	0.794	0.758
20	2.721	2.161	1.628	1.301	1.087	0.974
25	3.526	2.641	1.975	1.619	1.587	1.184

**Figure 5:**
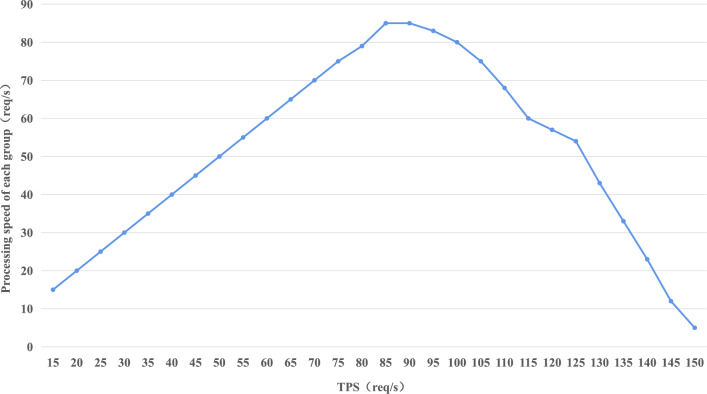
Each group’s processing speed under different TPS.

Directed Acyclic Graph (DAG) blockchain is the state-of-the-art solution for blockchain-based file transfer. It allows parallel and rapid processing of transactions and is designed for high performance. In contrast to traditional blockchains, which validate transactions and create new blocks at fixed time intervals, DAG blockchains offer a more efficient solution for real-time file transactions. For example, Ethereum commits blocks once every twelve seconds, limiting its ability to meet real-time transaction requirements. Both in our framework and DAG blockchains, each node can conduct transactions. However, unlike DAG blockchains, our framework incorporates a “central” group to enhance efficiency and security. It is important to note that while our framework achieves a higher degree of efficiency and security, DAG blockchains generally exhibit greater decentralization. To compare with the performance of DAG blockchains (e.g., IOTA), we refer to the experimental settings in other study^25^, the number of groups was increased to 30, each consisting of 8 nodes. We test average processing speed of each group from 15 to 150 TPS per second in 300 seconds. The result is shown in Fig. 5.

Among the three implementations of IOTA, Nano, and Byteball in the paper^25^, Nano can achieve a maximum throughput of 60 transactions per second. Our system can achieve 85 transactions per second for each group according to Fig. 5, and our global throughput needs to be multiplied by the number of groups. Compared to the state-of-the-art method, our method has made some progress.

## Conclusion

In this paper, to address the fundamental issue of file transfer, we propose a new blockchain-based framework for file transfer. To begin with, we propose the core functions of the entire system based on security requirements. Then, in order to efficiently complete the task, we design a dual-chain blockchain structure and new consensus based on the PBFT algorithm and sharding concept. Furthermore, we analyze the security, feasibility and efficiency of the framework. Finally, we conduct quantitative experiments in a simulated environment. The difference between our proposed framework and existing solutions lies in two aspects. Firstly, we adopt a relatively centralized consensus through the leadership group to ensure efficient operation of the system while ensuring security. The second is that transaction processing is highly parallel in each group, which solves the problem of low efficiency in existing blockchain file transfer solutions. But this framework is limited by the absence of practice under complex network, we haven’t designed a communication scheme for adverse network condition.

## Data Availability

The datasets generated and analyzed during the current study are available from the corresponding author on reasonable request.
